# Programmed cell death ligand 1 cut-point is associated with reduced disease specific survival in resected pancreatic ductal adenocarcinoma

**DOI:** 10.1186/s12885-017-3634-5

**Published:** 2017-09-05

**Authors:** Basile Tessier-Cloutier, Steve E. Kalloger, Mohammad Al-Kandari, Katy Milne, Dongxia Gao, Brad H. Nelson, Daniel J. Renouf, Brandon S. Sheffield, David F. Schaeffer

**Affiliations:** 1 Division of Anatomical Pathology, Vancouver General Hospital, Vancouver, British Columbia Canada; 2 Department of Pathology & Laboratory Medicine, University of British Columbia, Vancouver, British Columbia Canada; 3 Division of Medical Oncology, University of British Columbia , Vancouver, British Columbia Canada; 4 Genetic Pathology Evaluation Centre, University of British Columbia, Vancouver, British Columbia Canada; 5 Pancreas Centre BC, Vancouver, British Columbia Canada; 6 Deeley Research Centre, British Columbia Cancer Agency, Victoria, British Columbia Canada; 7 Division of Medical Oncology, British Columbia Cancer Agency, Vancouver, British Columbia Canada; 8 Department of Biochemistry and Microbiology, University of Victoria, Victoria, British Columbia Canada; 9 Department of Anatomical Pathology, Abbotosford Regional Hospital and Cancer Centre, Abbotsford, British Columbia Canada

**Keywords:** Pancreatic cancer, Programmed cell death 1 ligand, DNA mismatch repair, Tumor-infiltrating lymphocytes, Biomarkers, Immuno-oncology

## Abstract

**Background:**

Programmed cell death 1 (PD1) inhibitors have recently shown promising anti-cancer effects in a number of solid tumor types. A predictive biomarker to this class of drugs has not been clearly identified; however, overexpression of the PD1 ligand (PD-L1) has shown particular promise in lung adenocarcinoma. In this study, we explore the staining characteristics, prevalence, and clinico-molecular correlates of PD-L1 overexpression in pancreatic ductal adenocarcinoma (PDAC).

**Methods:**

A tissue microarray (TMA) was constructed from cases of resected PDAC. PD-L1 immunohistochemistry (IHC) was performed using the SP142 primary antibody. Immunohistochemical assessment for deficient mismatch repair status (MMRd), CD3 and CD8 were performed. All biomarkers were assessed independently by two anatomical pathologists and consensus achieved on all cases. Survival analysis was performed using three thresholds (> = 1%, >5% and >10%) for tumor cell membrane staining.

**Results:**

Two-hundred fifty-two cases were included in the TMA and evaluable by IHC. Thirty-one (12%), 17 (7%), 12(5%) cases were positive at percentage cut offs of >0, >5, and >10% respectively. Increased PD-L1 expression was associated with inferior prognosis (*p* = 0.0367). No statistically significant association was identified between PD-L1 status and MMR status or tumor infiltrating lymphocytes.

**Conclusions:**

This data suggests that there is an inverse relationship between PD-L1 expression and disease specific survival times in resected PDAC. Consequently, this association may represent a phenotype where increased PD-L1 expression has an effect on tumor biology and could therefore identify a subgroup where PD1 blockade could have enhanced effectiveness.

## Background

Pancreatic ductal adenocarcinoma (PDAC) ranks fourth for overall cancer-related death with over forty-thousand estimated deaths in 2015 in the United States. The five-year survival rate is 26% in resectable disease and drops to 2% if unresectable. Surgical resection is only attempted in 20% of cases [1].

Inhibitors of the programmed cell death 1 (PD1) signalling axis have yielded improved survival benefits for a number of solid tumor types. Large randomized clinical trials have been successful in treating melanoma, non-small cell lung cancer, and renal cell carcinoma [2, 3]. Three phase 1/2 drug trials are ongoing involving treatment of PDACs with immunotherapy (NCT02583477, NCT02305186, NCT02452424). To date, no biomarker has been established to predict benefit from PD1-axis inhibition for this disease [4].

PD-1 is an inhibitory receptor expressed by T cells and other immune cell types. It plays an important role in immune suppression when activated by its ligand (PD-L1). The latter is physiologically expressed by normal tissue and can occasionally be aberrantly expressed by tumor cells as a means for evading immune destruction [4–8]. Blockade of the PD-1/PD-L1 interaction promotes T-cell response against tumor cells [3, 9].

The response to PD-1/PD-L1 inhibition has been mixed in various malignancies such as colorectal, prostatic and pancreatic adenocarcinomas and is exemplified in the results of a study by Brahmer et al. which failed to show an objective response to anti-PD-L1 therapy in 14 patients with pancreatic cancer [3, 10]. In those cases, the use of biomarkers may have been useful in the identification of patients who are more likely to respond to PD1-axis inhibition. Mismatch repair (MMR) status has been shown to be predictive in colorectal carcinoma [11] and PD-L1 expression by immunohistochemistry (IHC) may be useful in lung and bladder carcinomas [12, 13]. However, no cut-off has been uniformly defined for PD-L1 expression that would trigger the use of PD-L1 inhibitors in PDAC. Current clinical trials often use 1% [14] but evidence suggests that higher cut-points may optimize patient stratification for PD-L1 therapies [15]. PD-1 expression in tumor infiltrating immune cells, the direct target of nivolumab, has shown, unlike tumor PD-L1 expression, only borderline association with clinical outcome to PD-1 blockade [16]. Other methods to predict response to immune checkpoint inhibitors have also been investigated, including immune cell infiltration, hypermutation signature, and gene expression linked to chemokine expression [17–19], but are yet to be validated in prospective clinical trials.

In this study, we explore the prevalence of PD-L1 expression in PDAC using IHC and compare this to clinical characteristics, including MMR status and tumor infiltrating lymphocytes and examine if an association with clinical outcome exists.

## Methods

Ethical approval and a waiver of consent for research on this retrospective cohort was obtained from the University of British Columbia Clinical Research Ethics Board (H12–03484).

### Sample identification and TMA construction

A tissue microarray was constructed using duplicate 0.6 mm cores from the epithelial component of all available, resected, pathologically confirmed pancreatic ductal adenocarcinomas derived from the archives of the Vancouver Coastal Health Region between 1995 and 2014. All patients received primary surgery with a subset receiving adjuvant chemotherapy with a pyrimidine nucleoside analog. Cores for the tissue microarray were obtained from areas of tumor as determined by routine microscopy on hematoxylin and eosin-stained sections. Cases were excluded if they lacked clinical follow-up data or if clinico-pathologic variables were lacking.

### Immunohistochemical staining of PD-L1 and mismatch repair markers

Immunohistochemistry was performed on 4-μm-thick formalin-fixed paraffin-embedded sections of tissue microarrays. PD-L1 immunohistochemistry was performed at the Deely Research Centre at the British Columbia Cancer Agency using the Intellipath FLX autostainer (Biocare) platform. Mismatch repair, CD3 and CD8 immunohistochemistry was performed in the clinical laboratory of Vancouver General Hospital using the Ventana Discovery XT and the Ventana Benchmark XT automated system (Ventana Medical Systems, Tucson, AZ).

For PD-L1, slides were incubated with the clone SP142 (Spring Bioscience, Pleasanton, USA) at 1/100 dilution in Da Vinci Green diluent at room temperature for 30 min. Slides were then washed and incubated with Mach2 Rabbit-HRP polymer for 30 min at room temperature and detected with IP DAB chromogen for 5 min. Nuclei were counterstained with a 1/10 dilution of CAT hematoxylin then slides were again washed, air dried and coverslipped with Ecomount. The antibody clone was selected based on its strong concordance to three other PD-L1 clones and RNA in situ hybridization (ISH) in NSCLC [19].

For MMR stains, slides were incubated with MLH1 (mouse monoclonal antibody, 1:50 dilution, cat#: NCL-L- MLH1, clone ID:ES05; Leica Microsystems, New- castle, UK), MSH2 (mouse monoclonal antibody, 1:1000 dilution, cat#: 286 M-16, clone ID:G219–1129; Cellmarque, Rocklin, CA), MSH6 (rabbit monoclonal antibody, 1:200 dilution, cat#: CLAC-0047, clone ID: EP49; Cedarlane Corporation, Burlington, ON, Canada), and PMS2 (rabbit monoclonal antibody, 1:20 dilution, cat#: CLAC-0049, clone ID:EP51; Cedarlane Corporation) for 32 min at room temperature. For the slides to be stained for PMS2 were additionally prepped with the Epitomics DAB prep kit before antibody incubation.

Antibodies were detected using the Ventana DABMap kit, counterstained with hematoxylin and treated with a proprietary bluing agent (Ventana). Positive and negative controls are performed as part of the routine clinical quality assurance; in addition to the external quality control program (Canadian Immunohistochemistry Quality Control (cIQc), a provider of proficiency testing for Canadian clinical laboratories).

### Interpretation of Immunohistochemical stains

PD-L1 status was assessed independently by two anatomical pathologists (BSS and DG) and consensus achieved on all cases. Positivity was evaluated by H-Score, a combination of staining intensity and percentage of tumor cell staining. Staining intensity was scored as 0 (negative), 1 (weak), 2 (moderate), or 3 (strong) based on membranous localization and each score multiplied by the percentage of cells (0% - 100%) staining. Therefore, H-scores ranged from 0 to 300. To account for potential intra-tumoral heterogeneity, the mean of both cores was used to generate the score for each case.

Mismatch repair (MMR) was quantified as per Riazy et al. [20]. Briefly, protein expression for MLH1, MSH2, MSH6, and PMS2 was considered intact (normal) if any percentage of definite positive nuclear staining of the malignant cells was detected on either TMA core. In cases where one or more mismatch repair proteins were interpreted as negative staining, examination utilizing immunohistochemistry on whole sections was performed. Each protein was considered lost (abnormal) if there was complete loss of nuclear staining in the tumor cells and if there was a positive internal control (intact nuclear staining of stromal elements such as inflammatory cells and/or endothelial cells) on whole section. Cases showing a complete absence of nuclear staining pattern of both tumor cells and stromal elements were deemed uninterpretable and thus excluded from the study. Cases that demonstrated loss of any MMR marker on the TMA were subjected to confirmatory whole slide section staining and were scored independently by two pathologists (BSS and DFS), who were blinded to clinical characteristics and patient outcomes. Divergent assessments were reconciled by consensus conference. A case was labeled as mismatch repair deficient (MMRd) if any of the four mismatch repair proteins was completely absent on immunohistochemistry. Cases were classified as mismatch repair proficient (MMRp) if all four proteins stained positive to some degree.

Individual tumor infiltrating lymphocytes were counted and typed in the epithelial and stromal compartments using clinically validated IHC stains for CD3 and CD8. Scoring was performed independently by two anatomical pathologists (BSS and MA-K) and consensus achieved on all cases. To account for potential intra-tumoral heterogeneity, the average of both cores were used to generate the final score for CD8+ and CD3+ tumor infiltrating lymphocytes.

### Clinico-pathologic variables and outcome

Standard treatment, clinical and pathologic parameters were collected from the British Columbia Cancer Agency which included: age at surgery, sex, adjuvant chemotherapy agents used, lymphovascular invasion, perineural invasion, pathologic primary tumor (pT) stage, and pathologic regional lymph-node status (pN). The primary outcome measure was defined to be disease-specific survival, where survival time was calculated as the difference between the date of last follow-up and the date of surgery, expressed in years. Patients were censored if they were alive at last follow-up or had died from a cause other than their pancreatic malignancy. Deaths attributable to treatment related toxicities or inter-current diseases were considered censored observations for this analysis.

### Statistical analysis

To determine if H-Score or the percent of positive cells for PD-L1 expression yield differential prognostic ability, each scoring method was subjected to an omnibus assessment utilizing the Cox-Proportional Hazards Model to determine if the expression of PD-L1 was a significant prognostic marker in the context of the clinico-pathologic variables outlined previously with the exception of pT-Stage due the fact that the vast majority of the cases in this cohort are pT3. The proportionality assumption for each variable was assessed through examination of Cox-Snell residuals and continuous variables were assessed for linearity. The PD-L1 scoring methodology with the smallest *P*-value was determined to have the strongest prognostic effect. Parametric survival analysis was used in order to further elucidate the gradient dependent effect of PD-L1 expression on disease specific survival (DSS) for the scoring methodology with the greatest prognostic effect. This procedure modelled the disease specific survival data with 5 different distributions which included: weibull, log-normal, exponential, frechet, and log-logistic. The best distribution to be used for parametric survival analysis was determined by selecting the one with the lowest Bayesian Information Criterion from the model fits. This analysis produces a quantile plot with logSurvival Time plotted against PD-L1 expression which illustrates the gradient dependent relationship between disease specific survival and PD-L1 expression. Based on these findings, a series of three cut-points were created starting with an H-Score or percentage positive cells of 1- as these identify the equivalent cases. Subsequent cut-points were set at increments of 5 which correspond to the increments used for the assessment of percent positive cells. The resultant groups were subjected to univariable survival analysis to quantify differences in disease specific survival using the Kaplan-Meier method. A multivariable approach to disease specific survival, using the Cox Proportional Hazards Model, was used to determine if survival differences between PD-L1 expression categories were independent of adjuvant chemotherapy. Assessment for heterogeneity of clinico-pathologic variables was performed with the following statistical approaches: continuous variables were examined using the Wilcoxon Rank-Sum Test, categorical comparisons were computed using Fisher’s exact test. A *P*-value of <0.05 was considered as statistically significant for all analyses. All analyses were computed with JMP v13.1 (SAS Institute, Cary, NC).

## Results

**Fig. 1:**
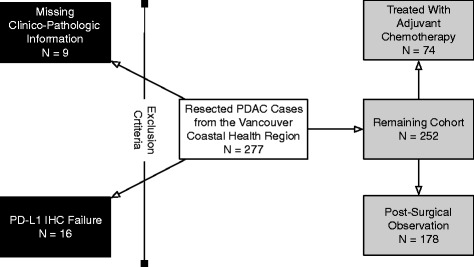
Patient selection diagram illustrating inclusion and exclusion criteria for this study with final numbers for the cohorts who received adjuvant pyrimidine nucleoside analogs or subjected to post-surgical observation only

After exclusion criteria were applied, two-hundred fifty-two cases remained (Fig. 1). The demographic information for the cohort were tabulated and are shown in Table 1. The Cox-Proportional Hazards Analysis for PD-L1 H-score and percent positive indicated that the latter was a stronger prognostic indicator with *P* = 0.0466 compared to H-Score with a *P* = 0.10 (Table 2).

**Table 1:** Demographics of the entire cohort

Variable	Levels	Values
Age	Median [IQR]	66.4 [13.3]
Sex	Male	139 (55.2%)
	Female	113 (44.8%)
Adjuvant Chemotherapy	Given	74 (29.4%)
	Observation	178 (70.6%)
Histologic Grade	1	2 (0.8%)
	2	186 (73.8%)
	3	64 (25.4%)
Lymphovascular Invasion	Present	144 (57.1%)
	Absent	108 (42.9%)
Perineurial Invasion	Present	232 (92.1%)
	Absent	20 (7.9%)
pT Stage	1	2 (0.8%)
	2	11 (4.4%)
	3	238 (94.4%)
	4	1 (0.4%)
pN Stage	0	64 (25.4%)
	1	168 (74.6%)
Resection Status	R0	190 (75.4%)
	R1	62 (24.6%)
CD3 Epithelial	Median [IQR]	0 [0]
CD3 Stromal	Median [IQR]	50 [52]
CD8 Epithelial	Median [IQR]	0 [0]
CD8 Stromal	Median [IQR]	11 [36]
MMR Status	Proficient	211 (84.1%%)
	Deficient	40 (15.9%)
Follow-up Time (Years)	Median [IQR]	1.33 [1.59]
Events	Disease Specific Deaths	200 (79.4%)
	Censorings	52 (20.6%)

**Table 2:** Multivariable disease specific survival analysis for PD-L1 expression quantified by percent positive & H-score

Variable	Comparison	Risk ratio	95%CI	*p*-value
PD-L1 H-Score				
PD-L1 H-Score	Per unit change	1.01	0.997–1.02	0.10
Age at Surgery	Per unit change	1.01	0.991–1.02	0.44
Sex	Male v Female	1.14	0.85–1.53	0.37
Adjuvant Chemotherapy	Given v Observation	0.59	0.42–0.81	0.0011
Histopathologic Grade	1 v 2	0.63	0.10–2.10	0.50
	1 v 3	0.49	0.08–1.67	0.28
	2 v 3	0.77	0.55–1.09	0.13
Lymphovascular Invasion	Present v Absent	1.27	0.93–1.74	0.13
Perineural Invasion	Present v Absent	1.66	0.96–3.09	0.07
pN-Stage	1 v 0	1.83	1.27–2.68	0.0010
Resection Status	R0 v R1	0.67	0.49–0.94	0.0202
PD-L1 Percent Positive				
PD-L1 Percent Positive	Per unit change	1.03	1.0005–1.05	0.0466
Age at Surgery	Per unit change	1.0006	0.99–1.02	0.42
Sex	Male v Female	1.16	0.86–1.55	0.33
Adjuvant Chemotherapy	Given v Observation	0.58	0.42–0.80	0.0008
Histopathologic Grade	1 v 2	0.64	0.10–2.13	0.51
	1 v 3	0.50	0.08–1.71	0.30
	2 v 3	0.78	0.56–1.10	0.15
Lymphovascular Invasion	Present v Absent	1.28	0.94–1.76	0.12
Perineural Invasion	Present v Absent	1.68	0.98–3.13	0.06
pN-Stage	1 v 0	1.85	1.28–2.71	0.0009
Rescetion Status	R0 v R1	0.68	0.49–0.95	0.0189

**Fig. 2:**
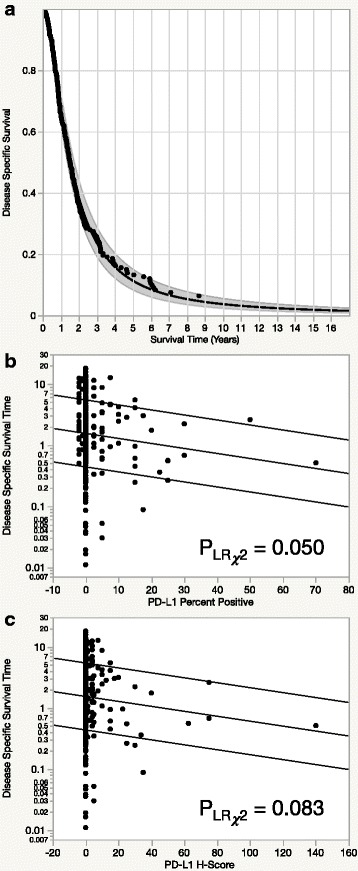
Parametric disease specific survival analysis using the log-logistic distribution to model disease specific survival in the entire cohort (**a**). Modeling of disease specific survival against PD-L1 expression assessed by percent positive (**b**) and H-Score (**c**) survival using a log-logistic distribution demonstrates a substantial association between reduced survival and increased PD-L1 expression. Curves shown are fitted as a function of the regressor representing the 0.9, 0.5, and 0.1 quantiles

Subjecting the survival data to the 5 distribution models outlined in the methods and ranking those fits by the Bayesian Information Criterion revealed that the log-logistic distribution fit best and was used as the basis for parametric disease specific survival analysis. Parametric disease specific survival of PD-L1 percent positive and H-Score demonstrated an inverse relationship between increased PD-L1 expression and survival time (Fig. 2). As indicated in the multivariable survival analysis, PD-L1 percent positive had a slightly stronger prognostic association compared to H-Score.

**Fig. 3:**
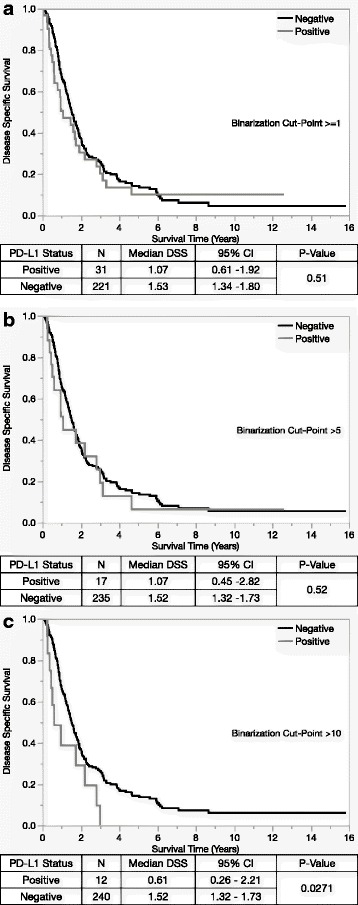
Binarization cut-points for percent positive (**a**-**c**) show that only the highest cut-point (>10) yields statistically significant survival differences

Based on the prognostic non-inferiority of PD-L1 percent positive, we elected to pursue this scoring method for the remainder of the study. Cut-points were determined according to our criteria outlined in the methods and resulted in: > = 1% (*N* = 31; 12.3% of the cohort), >5% (*N* = 17; 6.7% of the cohort), and >10% (*N* = 12; 4.8% of the cohort). Univariable survival analysis using these three cut-points showed no disease specific survival differences at the > = 1 cut-point (*p* = 0.51) or the >5 cut-point (0.52), but the >10 cut-point yielded statistically significant disease specific survival differences of *p* = 0.027 (Fig. 3). Multivariable DSS analysis of the >10% positive PD-L1 expression cut-point along with the other clinico-pathologic covariates outlined in Table 2, indicates that this subset of twelve cases has a trend toward inferior prognosis with a Risk Ratio and 95%CI 1.90 [0.96–3.42] (*P* = 0.06). When we sequentially removed statistically insignificant variables from the model (age, histopathologic grade, sex, and lyphovascular Invasion, PD-L1 > 10% became statistically significant Risk Ratio and 95%CI 2.05 [1.03–3.66] (*P* = 0.0410). The remaining statistically significant variables included pN-Stage (*P* < 0.0001), adjuvant chemotherapy (*P* = 0.0002), and perineurial invasion (*P* = 0.009) and resection status (*P* = 0.0263).

Analysis for heterogeneity across clinico-pathologic parameters which included: age, sex, adjuvant chemotherapy use, histopathological grade, lymphovascular invasion, perineural inavasion, pN-Stage, and resection status demonstrated a significant relationship between increased PD-L1 expression and higher grade cases (Table 3). The remaining clinico-pathologic variables, mismatch repair, and the stromal or epithelial compartment specific prevalence of CD3+ or CD8+ tumor infiltrating lymphocytes were not associated with the PD-L1 > 10% positive cells cut-point.

**Table 3:** Assessment for heterogeneity using a percent positive binarization cut-point of >10

Variable	Levels	PD-L1%Positive < 10	PD-L1%Positive > 10	*P*-Value
Age	Median [IQR]	66.5 [13.1]	65.6 [20.5]	0.96
Sex	Male	132 (55.0%)	7 (58.3%)	1.0
	Female	108 (45.0%)	4 (41.8%)	
Adjuvant Chemotherapy	Given	71 (29.6%)	3 (25.0%)	1.0
	Observation	169 (70.4%)	9 (75.0%)	
Histologic Grade	1	2 (0.8%)	0 (0.0%)	0.029
	2	181 (75.4%)	5 (41.7%)	
	3	57 (23.8%)	7 (58.3%)	
Lymphovascular Invasion	Present	136 (56.7%)	8 (66.7%)	0.56
	Absent	104 (43.3%)	4 (33.3%)	
Perineurial Invasion	Present	221 (92.1%)	11 (91.7%)	1.0
	Absent	19 (7.9%)	1 (9.3%)	
pT Stage	1	2 (0.8%)	0 (0.0%)	0.88
	2	10 (4.2%)	1 (8.3%)	
	3	227 (94.6%)	11 (91.7%)	
	4	1 (0.4%)	0 (0.0%)	
pN Stage	0	62 (25.8%)	2 (16.7%)	0.74
	1	178 (74.2%)	10 (83.3%)	
Resection Status	R0	184 (76.7%)	6 (50%)	0.08
	R1	56 (23.3%)	6 (50%)	
CD3 Epithelial	Median [IQR]	0 [0]	0 [4]	0.16
CD3 Stromal	Median [IQR]	50 [52]	63 [76]	0.52
CD8 Epithelial	Median [IQR]	0 [0]	0 [0]	0.51
CD8 Stromal	Median [IQR]	11 [36]	14 [39]	0.72
MMR Status	Proficient	200 (83.7%)	11 (91.7%)	0.70
	Deficient	39 (16.3%)	1 (8.3%)	

## Discussion

In this study, we have found a gradient dependent association between PD-L1 expression and inferior disease specific survival in resected pancreatic ductal adenocarcinoma. This finding was independent of the improved prognosis associated with the application of adjuvant chemotherapy with a pyrimidine nucleoside analog.

We assessed multiple scoring methods, H-Score or percent of positive cells, for the quantification of PD-L1 expression and determined that the estimation of percent positive cells yields a stronger association with inferior survival than H-Score. This suggests that the addition of a subjective intensity assessment to generate an H-score may represent an unnecessary step for the quantification of PD-L1 in this disease. Examination of other clinico-pathologic parameters revealed no statistically significant associations with PD-L1 expression at any cut-point, which indicates that PD-L1 expression does not select for any known prognostic variable other than histo-pathologic grade. Due to the limited power associated with our cohort combined with the small fraction of cases that express PD-L1 at a high level, we were limited in our ability to perform multivariable disease specific survival analyses with numerous variables. Exploratory multivariable disease specific survival modeling suggested that our categorized PD-L1 expression utilizing the cut-point of >10% of positive cells is independently associated with inferior disease specific survival and was only surpassed by the presence of regional lymph node metastasis and perineural invasion in terms of negative prognostic variables.

Recent studies have demonstrated that PD-L1 expression is associated with tumor types known to have higher somatic mutation load, as is the case for melanomas, NSCLC and RCC [21, 22]. Considering that PDAC has a lower mutation burden, it is not surprising that we found only 4 to 12% PD-L1 positive tumors compared to the reported 83% in melanoma, 50% in NSCLC and 80% in RCC [10]. Nonetheless, PDAC is associated with tobacco use and *BRCA* loss-of-function, and is predicted to, at least occasionally, show an increased mutation burden as a result of these [23]. Consequently, the lower than average rate of PD-L1 expression in PDAC compared to other malignancies may explain poor response to checkpoint inhibitors in clinical trials since PD-L1 was either not accounted for or the positivity thresholds were only set between 1% and 5% [10, 11]. Although our patient cohort was mostly treatment naive, we were able to identify differential outcomes based on higher PD-L1 expression.

The observed increased trend of lymphocyte tumor infiltration (CD3+) in PD-L1 positive patients has been reported in previous studies [24]. Sanmamed et al. showed that tumor infiltrating lymphocytes release IFN-Gamma as part of the host response to the tumor, which induces upregulation, and expression of, PD-L1 by tumor cells [25]. Our results indicate that a cut-point > = 1% yields the strongest association with CD3+ infiltrating T-cells but due to reduced power associated with increasing the PD-L1 cut-point, statistical significance is lost at higher thresholds.

We found no significant association between MMR and PD-L1 status. Our results are somewhat different from what was observed by Le et al. (2016) who reported that, in a series of 30 cases, PD-L1 was only expressed in MMR deficient (MMRd) tumors, most of which being colorectal carcinomas [11]. This inconsistency might be explained by the lower mutational burden seen in PDAC compared to MMRd colon carcinoma, melanoma, NSCLC and RCC [22]. Tumors with low mutational burden tend to be less immunogenic, making them less likely to develop immune silencing mechanism during their evolution.

There are several limitations to our study, one being the lack of consensus for PD-L1 IHC expression cut-off and gold standard, which our study has attempted to explore. Our IHC protocol for PD-L1 previously showed fairly strong concordance when compared to three other PD-L1 clones and RNA in situ hybridization (ISH), Sheffield et al., in NSCLC [26]. Our sample size is limited given the small percentage of PD-L1 expression and may have been underpowered to detect some more subtle associations, especially in the higher PD-L1 cut-points. Finally, since the IHC was performed on a TMA rather than full section, we might have underrepresented the amount of PD-L1 positive PDAC due to sampling error, although this method approximates the biopsy sampling error in encountered in clinical practice.

The prevalence of PD-L1 positivity in PDAC has been examined in numerous other studies with the percentage of tumor cells staining positive ranging from 4% - 49%. Each of these previous studies utilized different cut-points that varied between 1% - 10% making their results nearly impossible to compare [27–29]. Of particular interest, our results are somewhat different from what has been reported by Nomi et al. who demonstrated a found a 39% PD-L1 positivity in pancreatic cancer using a 10% positivity threshold [28]. Their cohort included 51 cases from Japan, which were stained using Anti-Human CD274, clone MIH1. The difference in PD-L1 expression is notable and although the CD274 is not commonly used in the clinical research setting this result may indicate variability associated with ethnicity.

## Conclusions

In summary, this is the first study to systematically investigate the association between clinical outcome and biomarker expression across differing scoring methodologies and cut-points for PD-L1 immunohistochemistry in this disease. We have demonstrated a gradient dependent association between PD-L1 expression and inferior survival that is independent of the prognostic advantage conferred by adjuvant chemotherapy. We postulate that the association presented here may indicate that higher PD-L1 protein expression levels represent a phenotype where PD-1 inhibition may be more effective. However, this hypothesis would have to be tested in the context of a randomized clinical trial. With studies in other diseases also indicating that deficient MMR (MMRd) status has been shown to be a predictive biomarker for immunotherapy, it is entirely plausible that PD-L1 immunohistochemistry is an imperfect biomarker for sensitivity to anti-PD-1 therapy. Interestingly, we found no association between MMRd status and PD-L1 expression in this cohort. More data on the role of PD-1-axis inhibition in PDAC is needed, specifically examining the use of predictive biomarkers in the context of patients treated with immunotherapy. Future studies should endeavor to build predictive models based on multi-marker expression that will serve as tools to triage the PDAC patient population to immunotherapy or other treatment regimens.

## Abbreviations

MMRd: Mismatch Repair Deficient
MMRp: Mismatch Repair Proficient
PD1: Programmed Cell Death 1
PDAC: Pancreatic Ductal Adenocarcinoma
PD-L1: Programmed Cell Death Ligand 1
